# Method optimisation to enrich small extracellular vesicles from saliva samples

**DOI:** 10.1002/ctm2.1341

**Published:** 2023-08-16

**Authors:** Abolfazl Jangholi, Juliana Müller Bark, Lucas Trevisan França de Lima, Luize Goncalves Lima, Andreas Möller, Lizbeth Kenny, Sarju Vasani, Sudha Rao, Riccardo Dolcetti, Chamindie Punyadeera

**Affiliations:** ^1^ The School of Environment and Science Griffith Institute for Drug Discovery (GRIDD) Griffith University Brisbane Queensland Australia; ^2^ Gallipoli Medical Research Foundation Greenslopes Private Hospital Greenslopes Queensland Australia; ^3^ Tumour Microenvironment Laboratory QIMR Berghofer Medical Research Institute Herston Queensland Australia; ^4^ Department of Otorhinolaryngology, Head and Neck Surgery, Faculty of Medicine The Chinese University of Hong Kong Sha Tin Hong Kong SAR; ^5^ Li Ka Shing Institute of Health Sciences The Chinese University of Hong Kong Sha Tin Hong Kong SAR; ^6^ Royal Brisbane and Women's Hospital Cancer Care Services Herston Queensland Australia; ^7^ Faculty of Medicine The University of Queensland Brisbane Queensland Australia; ^8^ Department of Otolaryngology Royal Brisbane and Women's Hospital Herston Queensland Australia; ^9^ Gene Regulation and Translational Medicine Laboratory QIMR Berghofer Medical Research Institute Brisbane Queensland Australia; ^10^ Peter MacCallum Cancer Centre Melbourne Victoria Australia; ^11^ Sir Peter MacCallum Department of Oncology The University of Melbourne Melbourne Victoria Australia; ^12^ Department of Microbiology and Immunology The University of Melbourne Melbourne Victoria Australia; ^13^ The University of Queensland Diamantina Institute Brisbane Queensland Australia; ^14^ Menzies Health Institute Queensland (MIHQ) Griffith University Gold Coast Queensland Australia

To the Editor:

Salivary small extracellular vesicles (sEV) contain cancer‐derived biomolecules, and sEVs can mediate cancer progression and metastasis.^1^ Given their biological roles during cancer pathogenesis,^2^ sEVs can be used as non‐invasive markers for disease diagnosis, prognosis, therapy selection and monitoring. However, our understanding of the influence of sEV isolation method on downstream analysis (e.g., proteomics) is limited. Here, we have evaluated four isolation methods for salivary sEVs, and compared them with plasma sEVs protocols.

Figure S1 depicts the flowchart of sEV isolation using saliva and plasma samples. We have compared size exclusion chromatography (SEC), ultracentrifugation (UC), ultracentrifugation plus filtration (UCF) and density gradient (DG) to isolate sEVs. In order to evaluate the yields of sEVs, the particle size and abundance were assessed using nanoparticle tracking analysis (NTA). Mean and mode sizes, the average and most frequent population of particle sizes, respectively, were determined. No significant variations were observed between means and modes of salivary sEVs (Figure 1A). For plasma sEVs, DG resulted in a significantly larger particle size than the UC and UCF (Figure 1B). In addition, sEVs with larger mean and mode sizes were detected in saliva in comparison to plasma (Figure S2A,B). DG and SEC resulted in the highest sEV yield for saliva and plasma, respectively (Figure 1C,D). Next, according to the number of particles per microgram of protein, calculated using a BCA method (Figure 1E,F), the purity of sEVs was assessed (Figure 1G,H). Overall, DG and SEC provided two‐ to six‐fold higher yields and purity of sEVs derived from saliva and plasma, respectively.

**FIGURE 1:**
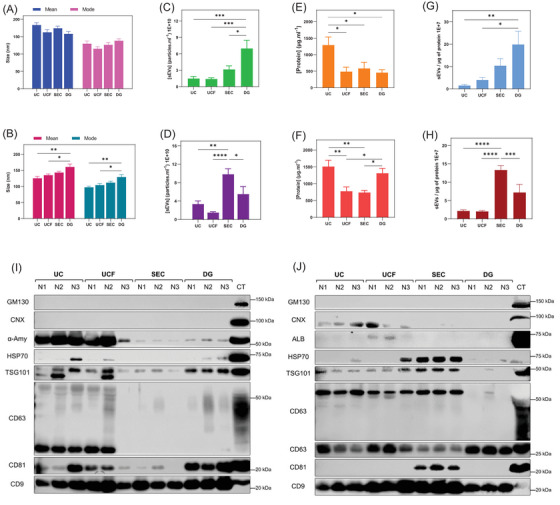
Comparison of sEVs in saliva (A, C, E and G) and plasma (B, D, F and H) samples isolated by different methods. The influence of isolation method on the size (A and B), total number (C and D), protein concentrations (E and F) and purities of sEVs (G and H). Data are presented as *n* = 8 ± SEM; *p < .05, **p < .01, ***p < .001, ****p < .0001. Western blot analysis for sEV and non‐sEV markers in saliva (I) and plasma (J) samples isolated by different methods. Seven micrograms of protein for each isolation method using saliva and plasma samples (N1, N2 and N3) was separated and then blotted in the presence of different antibodies. Cleared saliva and plasma plus cell lysate were used as control (CT). CNX: calnexin; ALB: albumin; α‐Amy: α‐amylase; UC: ultracentrifugation; UCF: ultracentrifugation plus filtration; SEC: size exclusion chromatography; DG: density gradient; sEVs: small extracellular vesicles.

To further assess the purities of the isolated sEVs, sEV and non‐sEV markers as well as alpha‐amylase (α‐Amy) and albumin (ALB) as the most abundant saliva and plasma proteins, respectively, were analysed using Western blot. Among all methods, DG method could isolate relatively purer salivary sEVs (positive for a battery of sEV markers CD9, CD63, CD81, TSG101 and Syntenin‐1) (Figure 1I and Figure S2C). In contrast, plasma samples isolated by SEC (Figure 1J and Figure S2FC) were enriched for all positive sEV markers (CD9, CD63, CD81, HSP70, TSG101 and Syntenin‐1). Some markers were either weakly or not detected in sEVs isolated using the other methods, which can be due to below reasons. First, this may be due to the distinct sEV subtypes isolated by each method.^3^ Second, it may be due to enrichment of non‐sEV lipid particles and/or empty vesicles.^4^ Third, some markers are cell‐type specific and not released in large quantities into biofluids such as saliva and blood.^5^ The presence of α‐Amy and ALB in samples isolated by UC and UCF indicates that the absence of cell organelle markers is useful, but not sufficient, to rule out other protein contaminations.

Next, the size, morphology and integrity of sEVs were evaluated using transmission electron microscopy (TEM) (Figure 2 and Figure S3). Salivary sEVs isolated by UC, UCF and SEC demonstrated particle clustering in some regions (Figure S3A–C). The tendency of sEVs (yellow arrows) to aggregate was more evident in samples isolated using UC than those isolated by other methods (Figure S3A7–10). In contrast, for plasma samples, UC and UCF displayed average sizes of sEVs (Figure S3E,F). Also, protein clusters and/or cell debris were observed in UCF (blue arrows). SEC and DG showed a range of small to large EVs, with protein aggregates and cell debris only found in DG (Figure S3G,H). Furthermore, saliva‐derived sEVs were dispersed, larger and fewer than plasma, which can be partly explained by their properties. While salivary glands, either ductal or acinar cells, have been mainly implicated in secretion of sEVs, the origin of sEVs in human plasma is largely a mixture of components derived from circulating immune cells.^6^, ^7^ Overall, TEM demonstrated that isolated particles display the expected characteristics of sEVs with a cup‐shaped morphology and a heterogeneous size ranging from approximately 40 to 200 nm, but in the presence of some contaminants such as proteins, other particles and/or cell debris.

**FIGURE 2:**
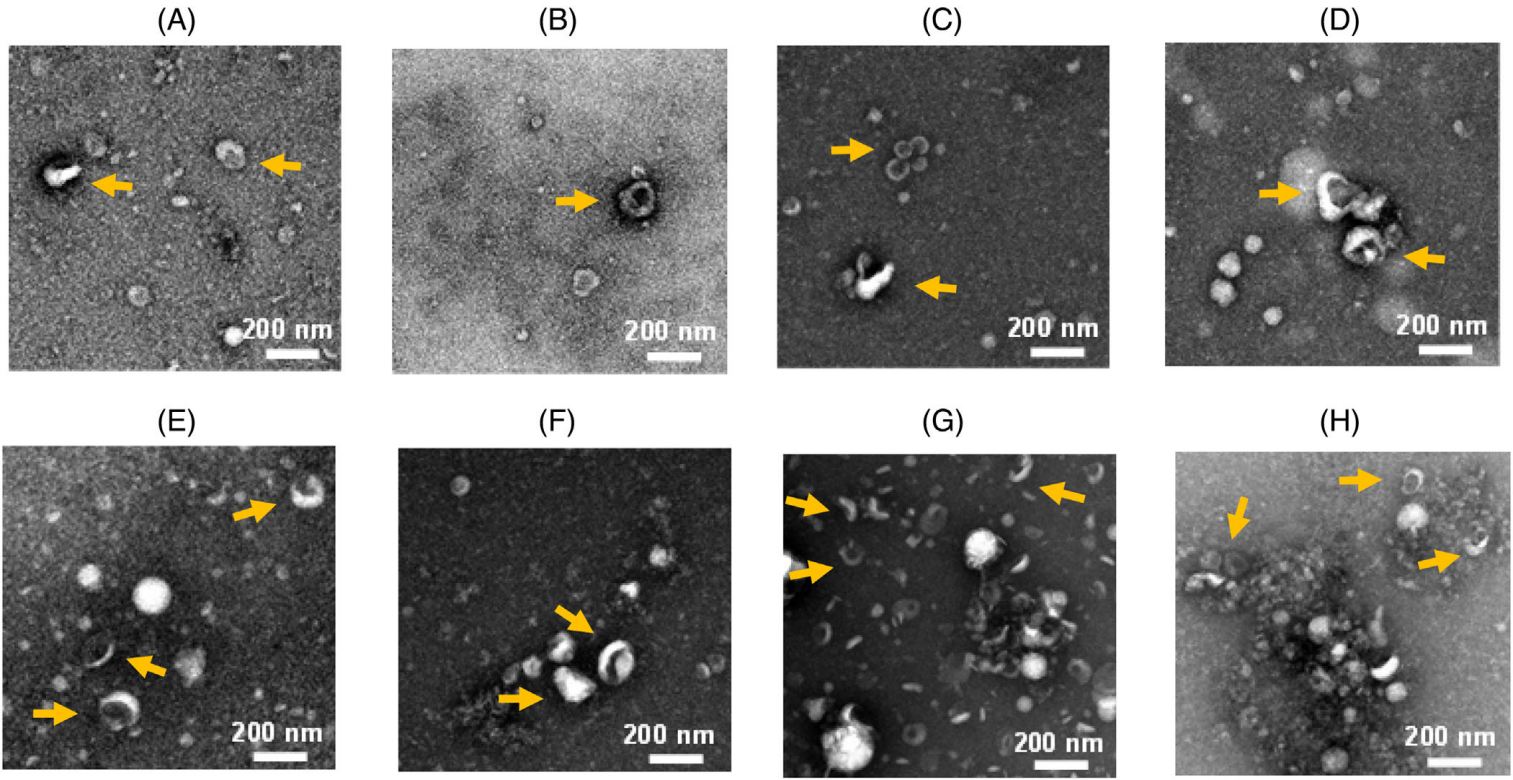
Characterisation of sEVs isolated from saliva (A–D) and plasma (E–H) samples by different methods using transmission electron microscopy (TEM). TEM confirmed the presence of sEVs with a cup‐shaped morphology (yellow arrows) and a heterogeneous size ranging from 40 to 200 nm, but in the presence of some contaminants such as proteins, other particles and/or cell debris. sEVs isolated by UC (A and E), UCF (B and F), SEC (C and G) and DG (D and H). UC: ultracentrifugation; UCF: ultracentrifugation plus filtration; SEC: size exclusion chromatography; DG: density gradient; sEVs: small extracellular vesicles.

To investigate the protein composition of sEVs isolated by the four methods and to determine the optimal method, MS analysis was performed (Figure S4). A complete list of proteins identified in each protocol can be found in Table S1. The number of common and unique proteins identified using each protocol for saliva and plasma appears in Figure 3A,B. As mentioned before, each method can specifically separate different sEV subpopulations with specific protein profiles, indicating that isolation methods could introduce bias in sEV populations. Therefore, we can infer that the choice of sEV isolation method has a direct impact on protein content. We have also found that there is a 17%–25% overlap of the sEV proteins between saliva and plasma samples (Figure 3C). The highest overlap of salivary and plasma sEV proteins was identified between UC and SEC techniques (141/25.6%). Furthermore, we have also compared the sEV proteins identified in each method, with the top 100 EV proteins and all the proteins listed in Vesiclepedia database^8^, ^9^ (Figure 3D). In saliva sEVs, UC (63) and DG (54) showed a higher representation of the top 100 EV proteins when compared to UCF (45) and SEC (53). UC (347) and DG (301) also led to the isolation of higher sEV proteins previously reported in the Vesiclepedia database. In contrast, for plasma‐derived sEVs, UC, UCF and DG displayed 22, 21 and 20 common proteins, respectively, when compared to the top 100 EV proteins, whilst SEC showed 52 common proteins. Similarly, SEC showed a higher number of overlapping proteins (287) compared to the Vesiclepedia database. Taken together, the proteins detected in sEVs derived from saliva and plasma can be vastly different depending on the isolation method. It should be noted that the choice of isolation method can significantly affect the success of enrichment for biomarker identification. Thus, selecting optimised sEV‐enrichment method in designing biomarker studies is of great importance.

**FIGURE 3:**
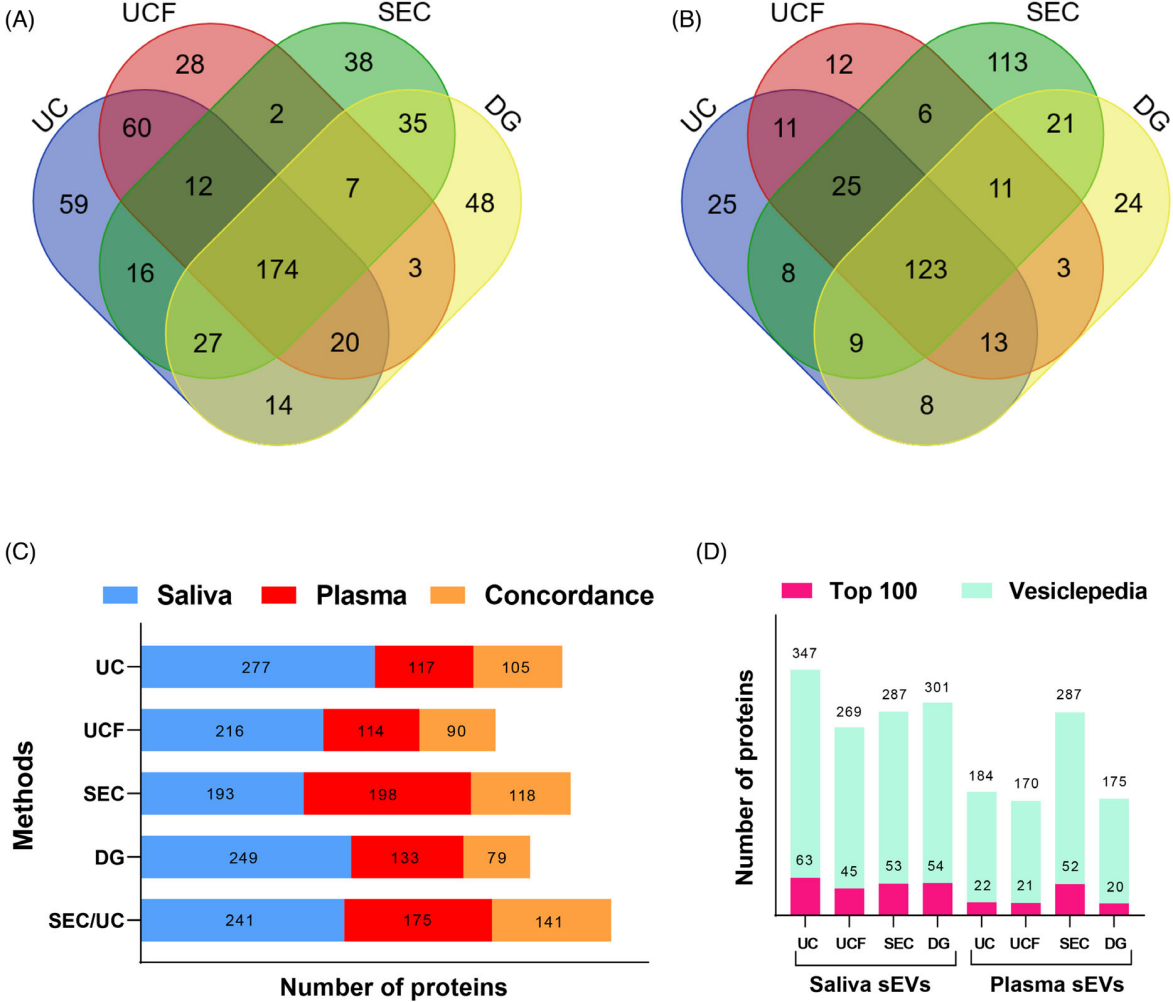
Number of identified proteins for each method using sEVs derived from four pooled saliva and plasma samples (*N* = 2). Venn diagram comparing common and unique proteins identified in sEV samples derived from saliva (A) and plasma (B) isolated by UC, UCF, SEC and DG. Number of common salivary and plasma sEV proteins identified in each protocol (C). Number of top 100 EV and all Vesiclepedia proteins identified in salivary and plasma sEV samples using the four methods (D). UC: ultracentrifugation; UCF: ultracentrifugation plus filtration; SEC: size exclusion chromatography; DG: density gradient; sEVs: small extracellular vesicles. Venn diagrams were produced using Bioinformatics & Systems Biology website (http://bioinformatics.psb.ugent.be/webtools/Venn/).

Table 1 compares the advantages and disadvantages of protocols used in this study for salivary and plasma sEV isolation. The low particle recovery of ultracentrifugation‐based methods (UC and UCF) may be due to damage and particles loss during repeated ultracentrifugation and washing steps. The utilisation of 0.2‐μm filters can lead to the reduction of particles due to the deformation and breaking‐up of sEVs and/or clogging the pores of filter. DG is a well‐known technique that provides the highest degree of purity in saliva‐ and plasma‐derived sEV to achieve accurate analyses of EVs. However, it requires both technical knowledge and a long centrifugation process. Although SEC was able to isolate plasma sEVs with high yield and purity, as confirmed by NTA and WB, it was not suitable for salivary sEVs isolation. This can be explained by the inherent viscoelasticity properties of saliva (5–7 cP), which makes it more challenging than plasma (1.2–1.3 cP).^10^

**TABLE 1:** Comparison of four protocols for salivary and plasma sEV isolation.

Method	Yield	Purity	Protein yield	Proteome yield	Ease of use	Hands‐on time	Turnaround time
S‐UC	✽	✽	✽✽	✽✽✽✽	✽✽	1	3
S‐UCF	✽	✽✽	✽✽	✽✽	✽✽	1	3
S‐SEC	✽✽	✽✽✽	✽	✽✽✽	✽✽✽✽	1	1
S‐DG	✽✽✽✽	✽✽✽✽	✽✽✽	✽✽✽	✽	2	20
P‐UC	✽✽	✽	✽✽	✽✽	✽✽	1	5
P‐UCF	✽	✽	✽✽	✽✽	✽✽	1	5
P‐SEC	✽✽✽✽	✽✽✽	✽✽✽✽	✽✽✽✽	✽✽✽✽	1	1
P‐DG	✽✽✽	✽✽	✽	✽✽	✽	2	20

*Note*: sEVs yield (based on NTA), purity (based on NTA and WB), protein yield (based on WB), proteome yield (based on number of identified proteins and overlapping proteins with Vesiclepedia), ease of use (based on the required time, technical knowledge and instrumental complexity). ✽ = Low; ✽✽ = moderate; ✽✽✽ = high; ✽✽✽✽ = very high.

Abbreviations: DG, density gradient; NTA, nanoparticle tracking analysis; P, plasma; S, saliva; SEC, size exclusion chromatography; sEV, small extracellular vesicle; UC, ultracentrifugation; UCF, ultracentrifugation plus filtration; WB, Western blot.

The main drawbacks of our study relate to a small sample size and the lack of functional analysis of sEVs. Future studies should incorporate functional assays using a large cohort of participants to investigate the specific functions and mechanisms of action associated with isolated sEVs. This will contribute to a deeper understanding of the biological relevance of sEVs and facilitate their translation into clinical applications.

In summary, we found that the appropriate sEV isolation method is vital for downstream analysis, especially during the discovery phase. DG and SEC methods resulted in higher yields and purity for salivary and plasma sEVs, respectively, making them more reliable for sEV‐based proteomic and biomarker discovery.

## CONFLICT OF INTEREST STATEMENT

The authors declare they have no conflicts of interest.

## Supporting information

Supporting Information

Supporting Information
