# Supplementary material for: Method optimisation to enrich small extracellular vesicles from saliva samples
Source: Clin Transl Med. 2023 Aug 16;13(8):e1341. doi: 10.1002/ctm2.1341 (PMC10432497; doi:10.1002/ctm2.1341)
Supplement: Supplementary file 1 — Supporting Information [file CTM2-13-e1341-s002.docx]

**Supporting Information**


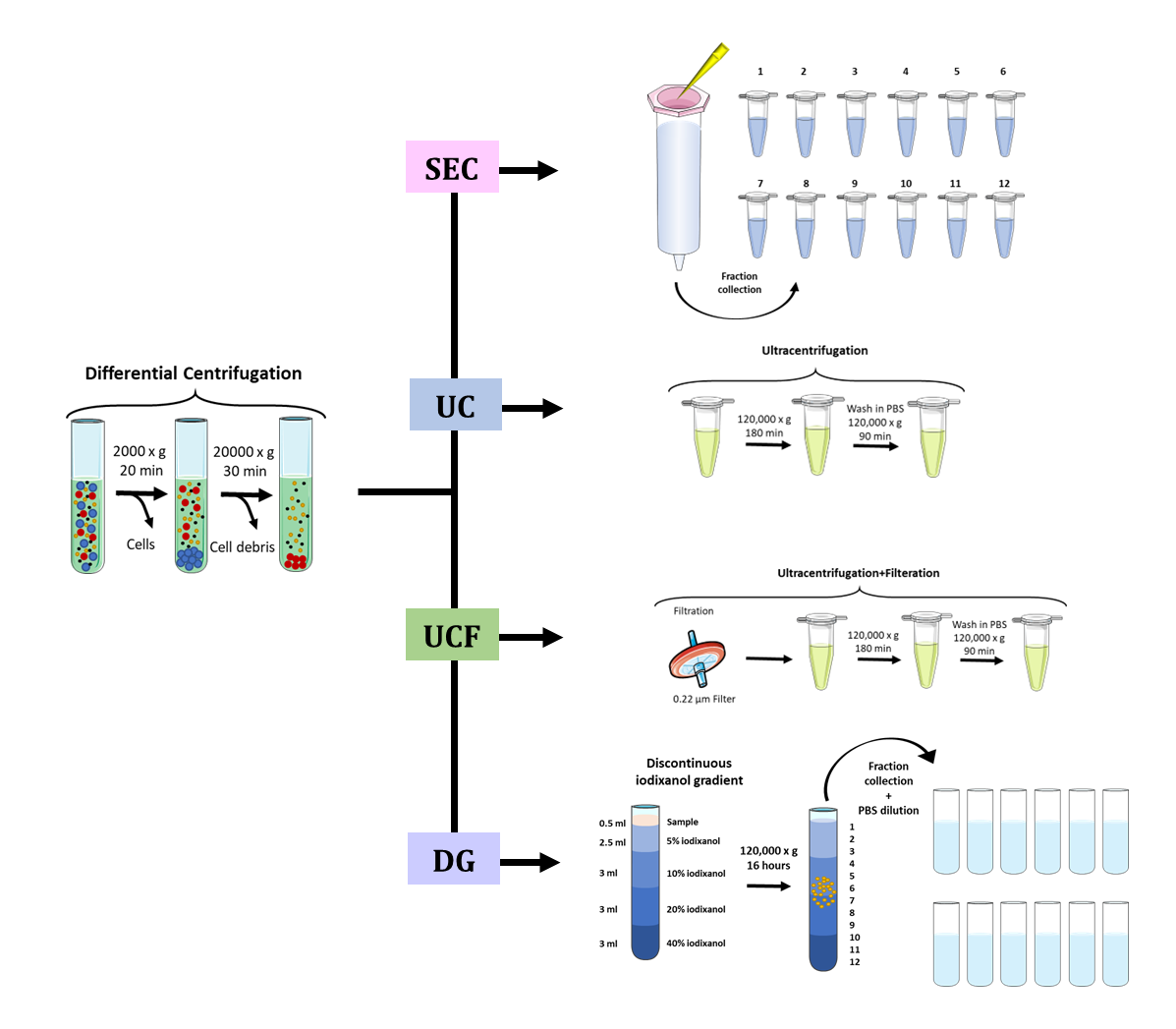


Figure S1. The flowchart representing sEV isolation from saliva and plasma using size exclusion chromatography (SEC), ultracentrifugation (UC), ultracentrifugation plus filtration (UCF) and density gradient (DG). Saliva and blood samples were obtained from eight healthy adult volunteers. The collection was performed in the morning, between 9 and 11 am. Volunteers were asked to donate saliva using a passive drool method. Following blood collection in ethylenediaminetetraacetic acid (EDTA) tubes, the samples were spun at 500 ×g for 15 minutes at room temperature. Saliva and plasma samples were then aliquoted and used for the isolation step. To remove dead cells, apoptotic bodies and large vesicles, all saliva and plasma samples were centrifuged at 2000×g for 20 min and then at 20,000×g for 30 min at 4°C. These samples are hereafter referred to as ‘clarified saliva’ and ‘clarified plasma’. SEC was carried out using qEV original / 70 nm Gen 2 size exclusion columns (Izon) under gravitational force. After loading clarified saliva and plasma (0.5 mL) onto the column, 12 fractions of 1 mL were collected and concentrated. All the fractions were analyzed for CD9, CD63 and Calnexin using western blot. Positive fractions for CD9 and CD63 were considered as isolated sEVs, thus pooled, concentrated and stored at -80 °C before use. UC and UCF were performed using one and two-step ultracentrifugation, respectively, for saliva and plasma samples, at 120,000×gavg at 4°C for 180 min and then for 90 min. For UCF, samples were filtered with 0.20 μm Syringe Filters before ultracentrifugation. DG was performed using a discontinuous gradient of 40%, 20%, 10% and 5% w/v iodixanol solutions. The clarified saliva and plasma samples (0.5 mL) were overlaid on the top of the gradient and spun for 16 h at 100,000 ×g avg at 4°C. After the spin, the gradients were fractionated from the top (12 fractions of 1 mL), diluted with ice-cold PBS (20 mL) and spun for 100 min at the same speed. The pellets were resuspended in 100 µL PBS and analyzed for CD9, CD63 and Calnexin using western blot. Positive fractions for CD9 and CD63 were considered as sEVs, thus pooled, concentrated and stored at -80 °C before use. This figure was produced using Servier Medical Art templates, licensed under a Creative Commons Attribution 3.0 Unported License; <https://smart.servier.com>.

|   A |   B |
| --- | --- |
| 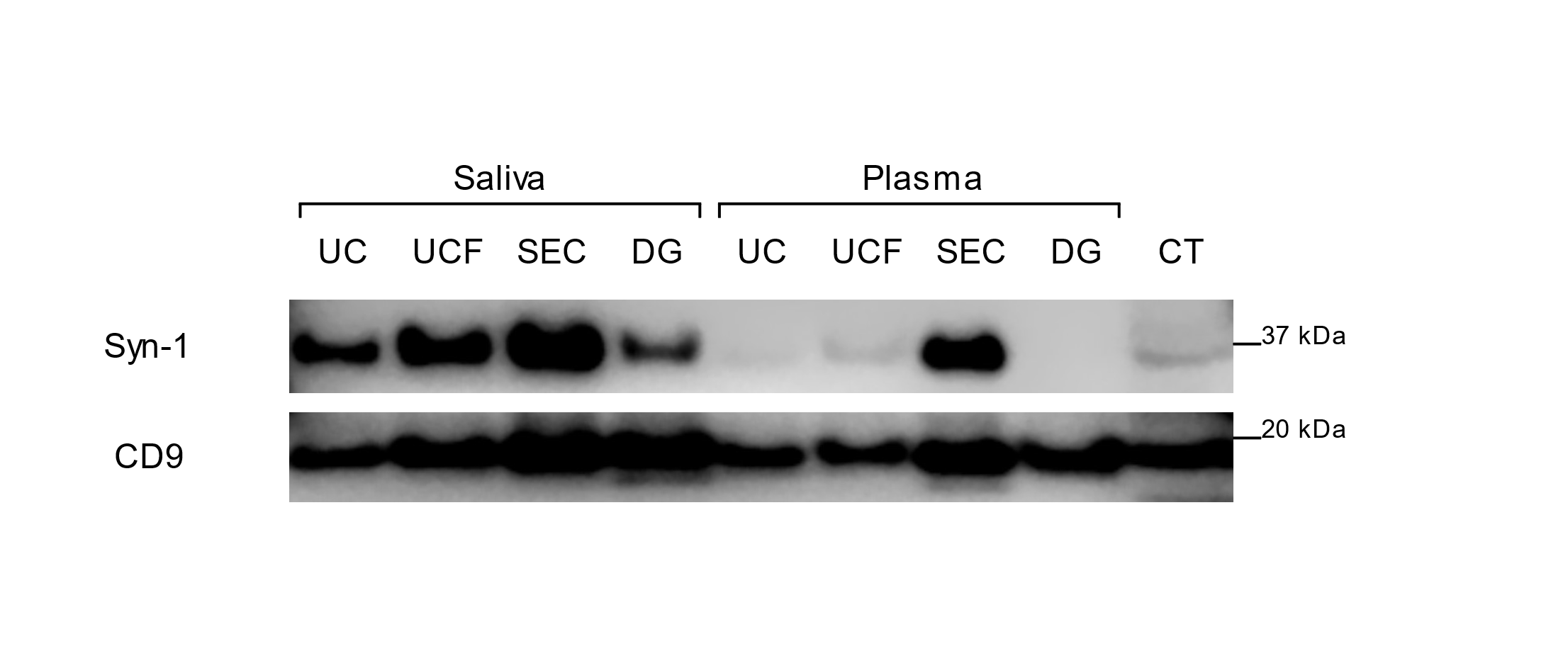  C | |

Figure S2. Mean (A) and mode (B) sizes of sEVs isolated from saliva and plasma by different methods. Data are presented as n = 8 ± SEM; * p<0.05, ** p<0.01, *** p<0.001, **** p<0.0001. Western blot analysis (C) for CD9 and Syn-1 in saliva and plasma samples isolated by different methods. 15 micrograms of protein for each isolation method using pooled saliva and plasma samples were separated and then blotted in the presence of different antibodies. Cell lysate was used as control (CT). S: saliva; P: plasma; UC: ultracentrifugation; UCF: ultracentrifugation plus filtration; SEC: size exclusion chromatography; DG: density gradient; Syn-1: Syntenin-1.

| 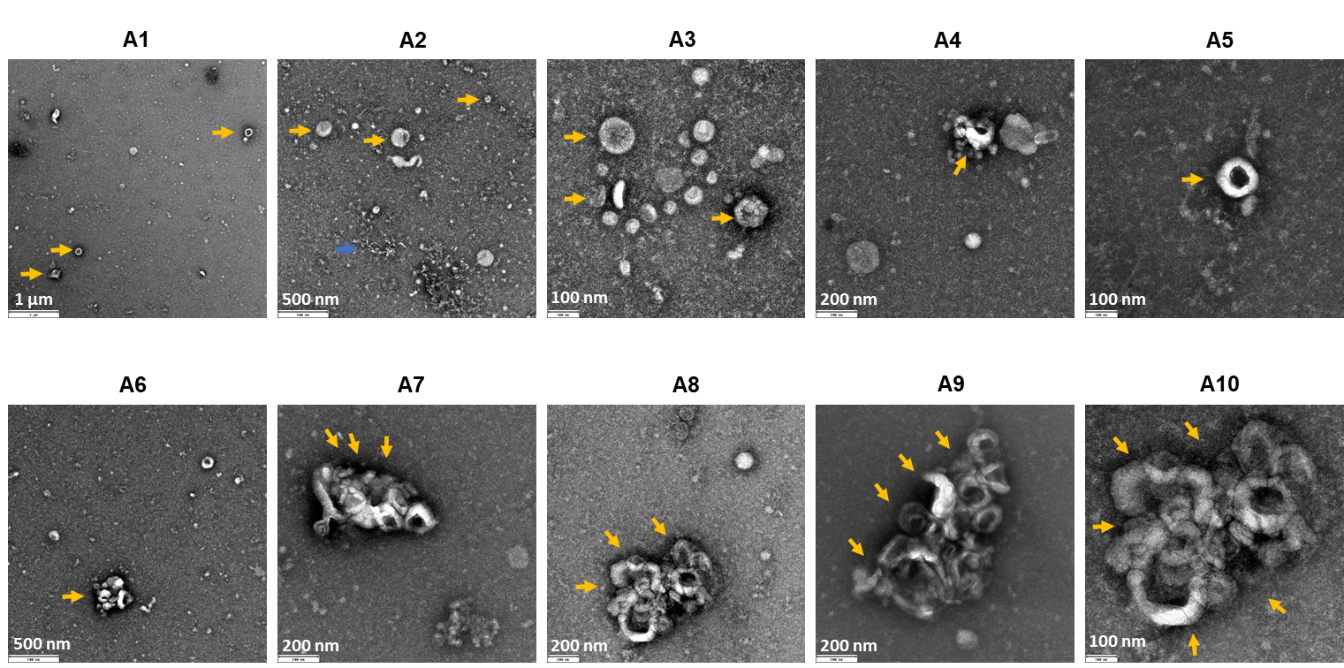 |
| --- |
|  |
| 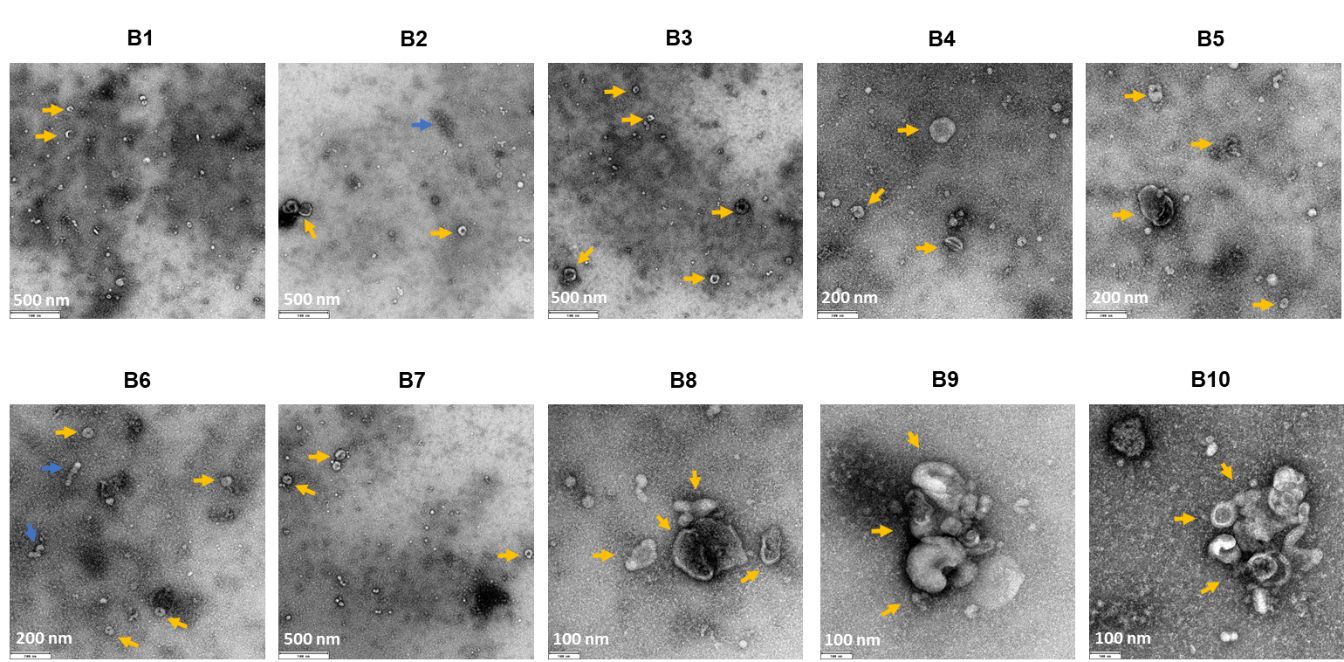 |
|  |
| 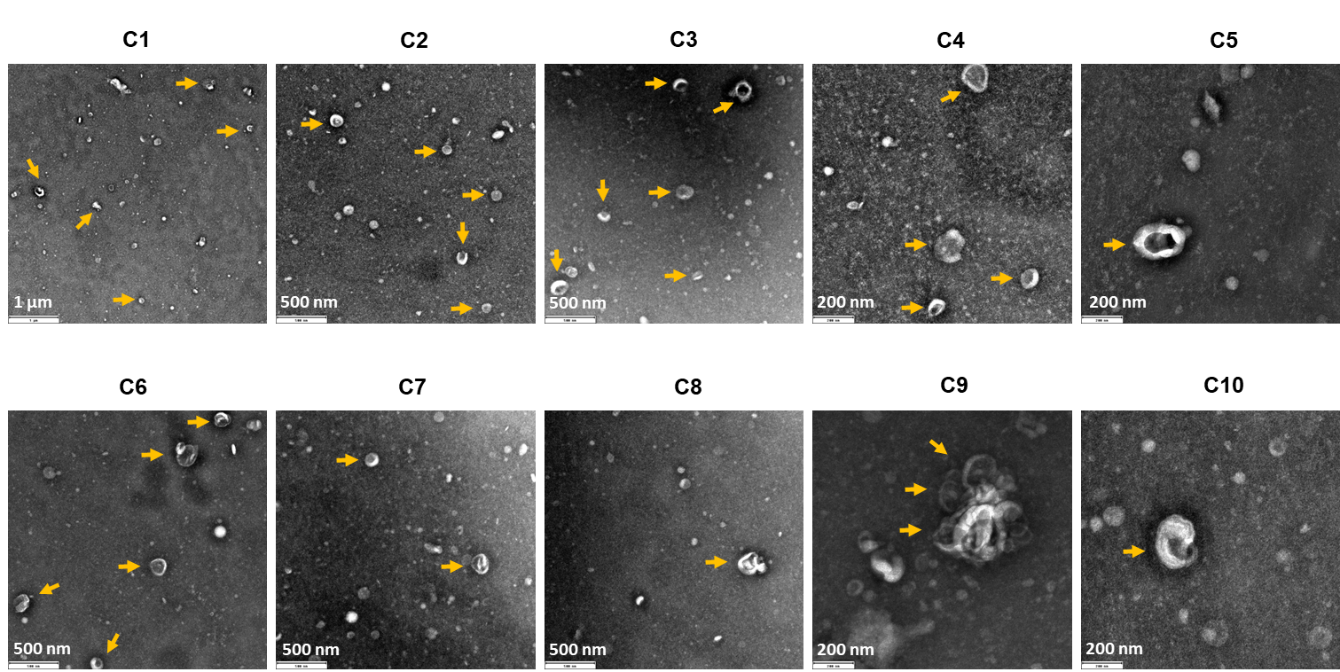 |
|  |
| 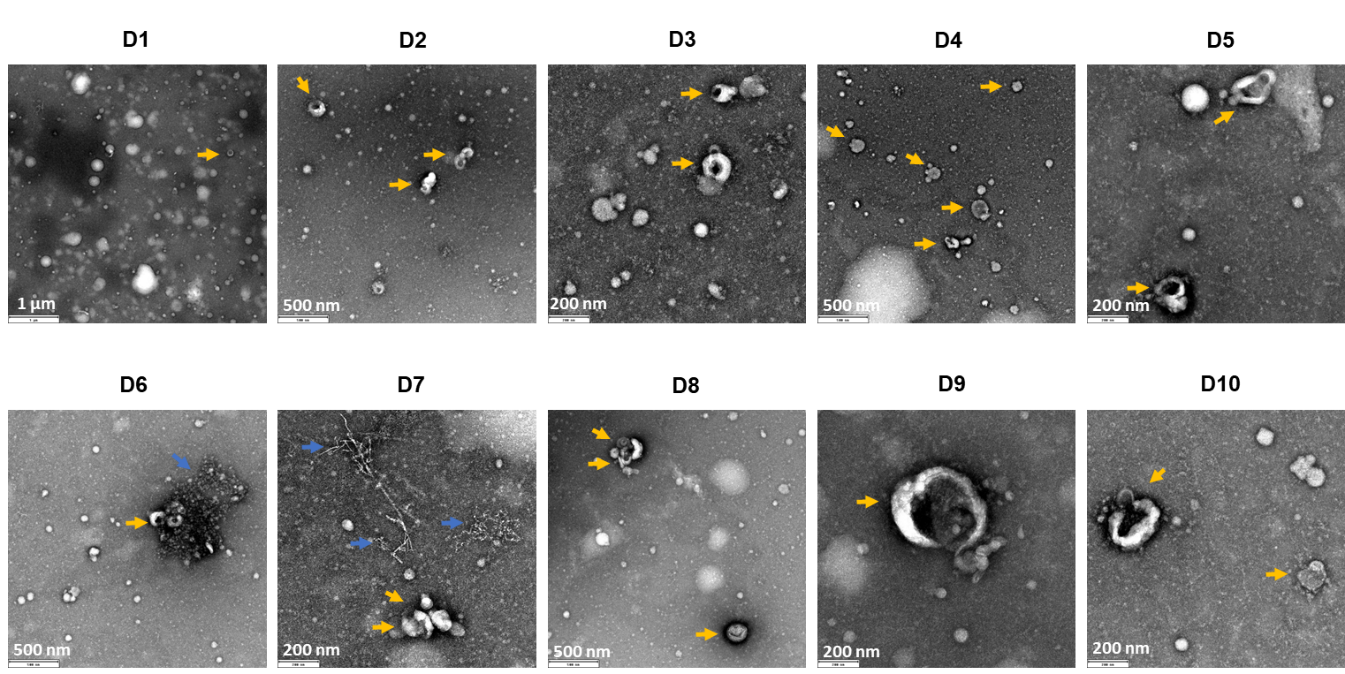 |
|  |
| 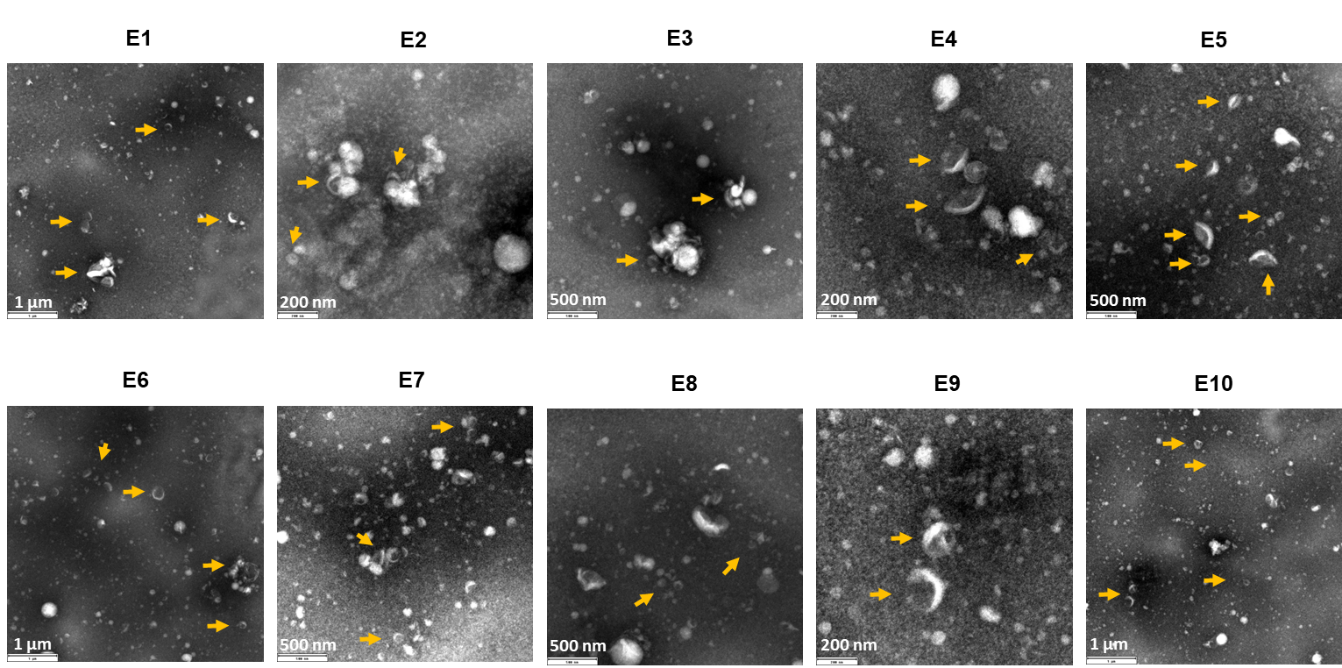 |
|  |
| 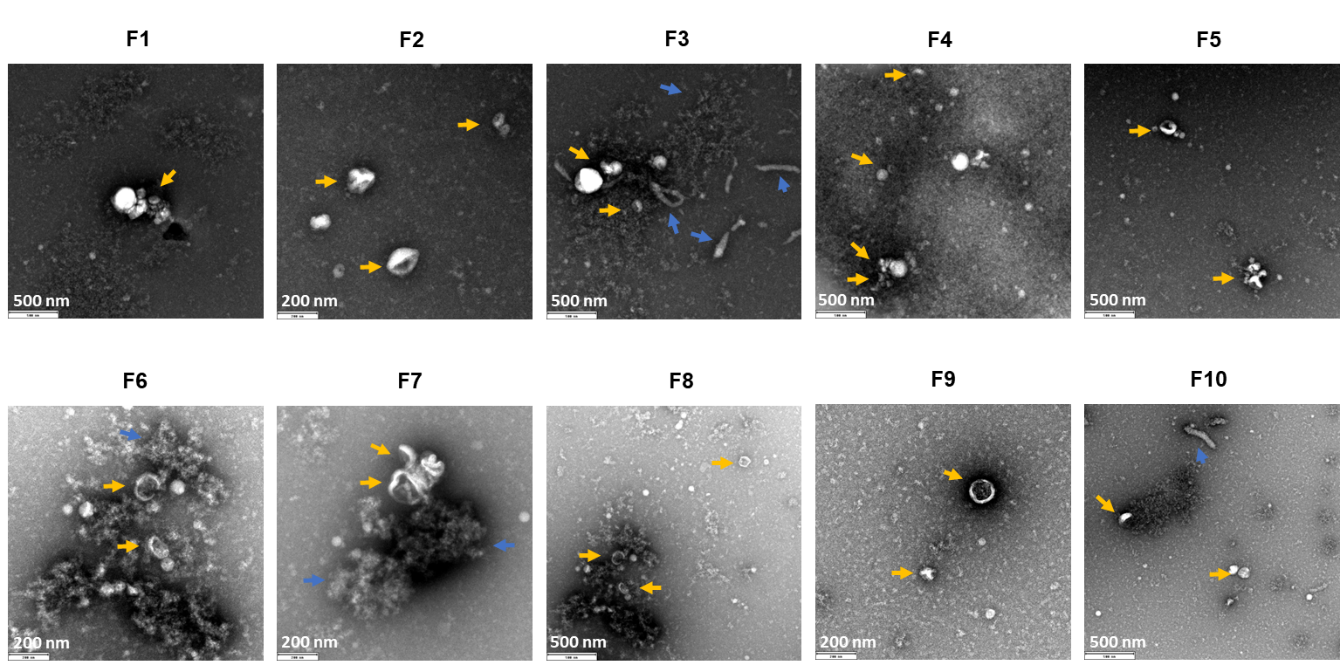 |
|  |
| 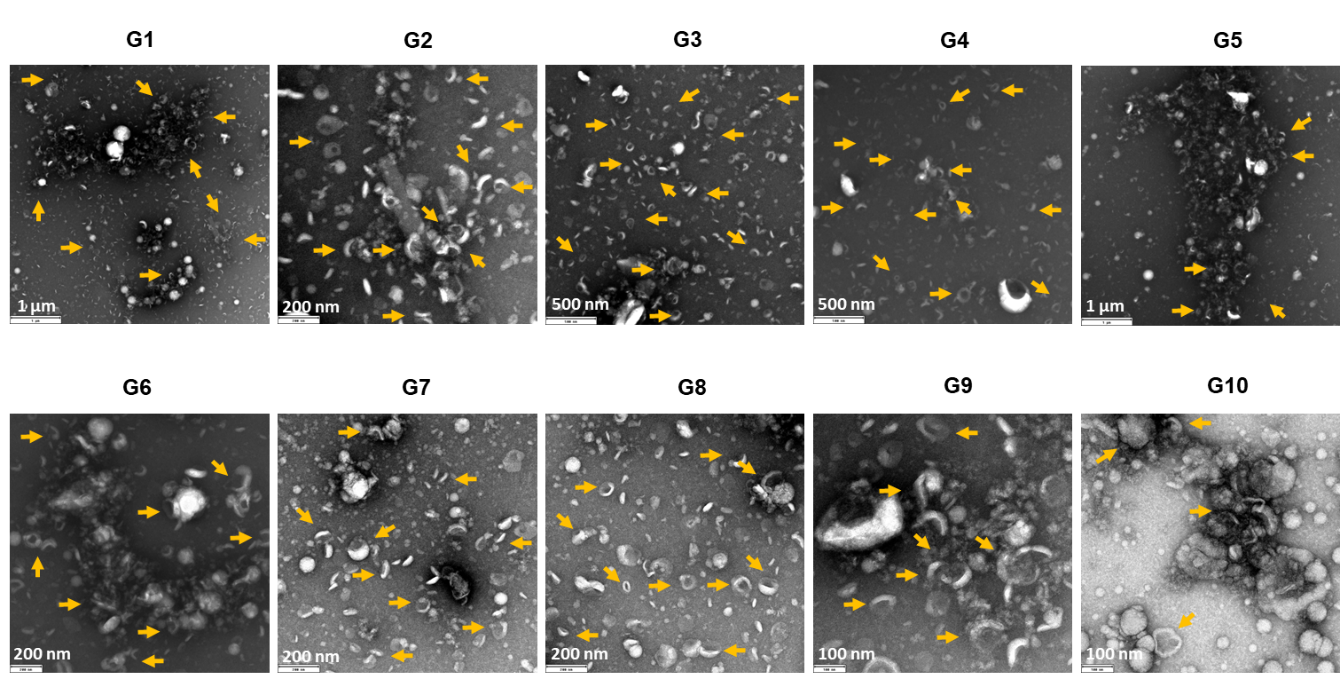 |
|  |
| 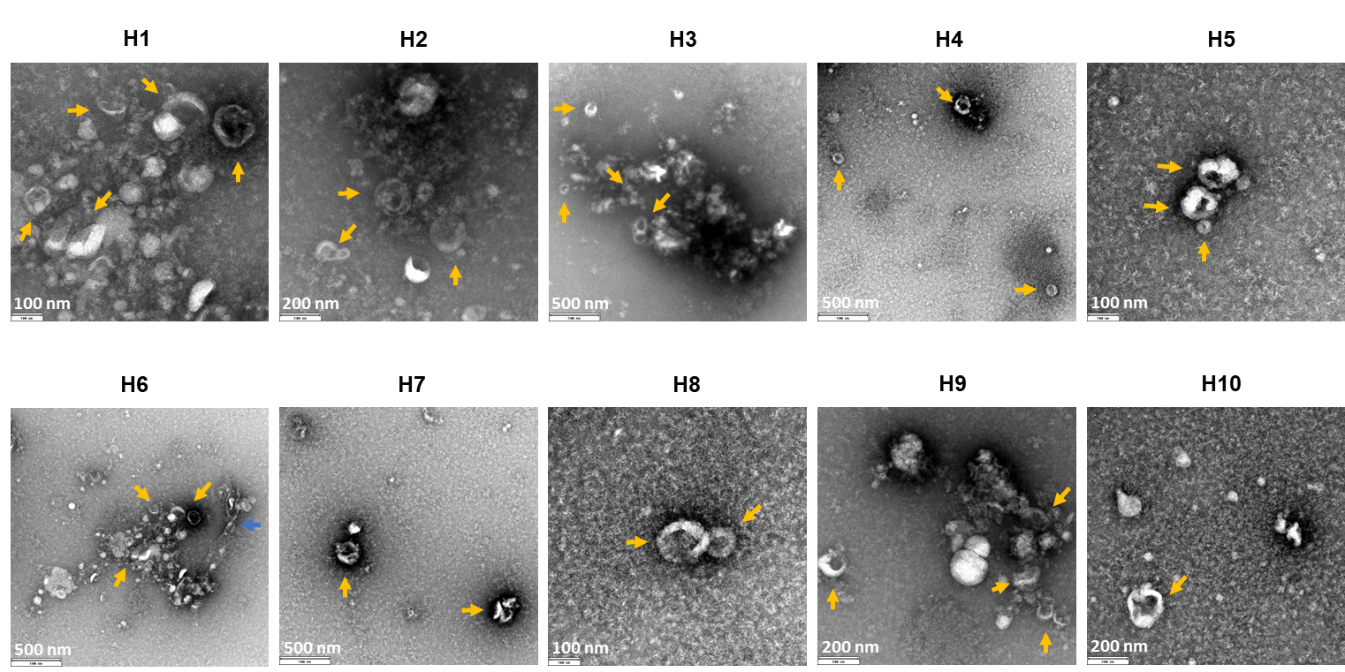 |

Figure S3. Characterization of sEVs isolated from saliva (A-D) and plasma (E-H) samples by different methods using transmission electron microscopy (TEM). Salivary sEVs isolated by UC (A), UCF (B), SEC (C) and DG (D) were fewer in number, larger in size, and more clustered together compared to those isolated from plasma. Plasma sEVs isolated by UC (E), UCF (F), SEC (G) and DG (H) were smaller, more numerous, and more uniformly separated. TEM confirmed the presence of sEVs with a cup-shaped morphology (yellow arrows) and a heterogeneous size ranging from 40 to 200 nm, but in the presence of some contaminants such as proteins, other particles and/or cell debris (blue arrows). UC: ultracentrifugation; UCF: ultracentrifugation plus filtration; SEC: size exclusion chromatography; DG: density gradient.


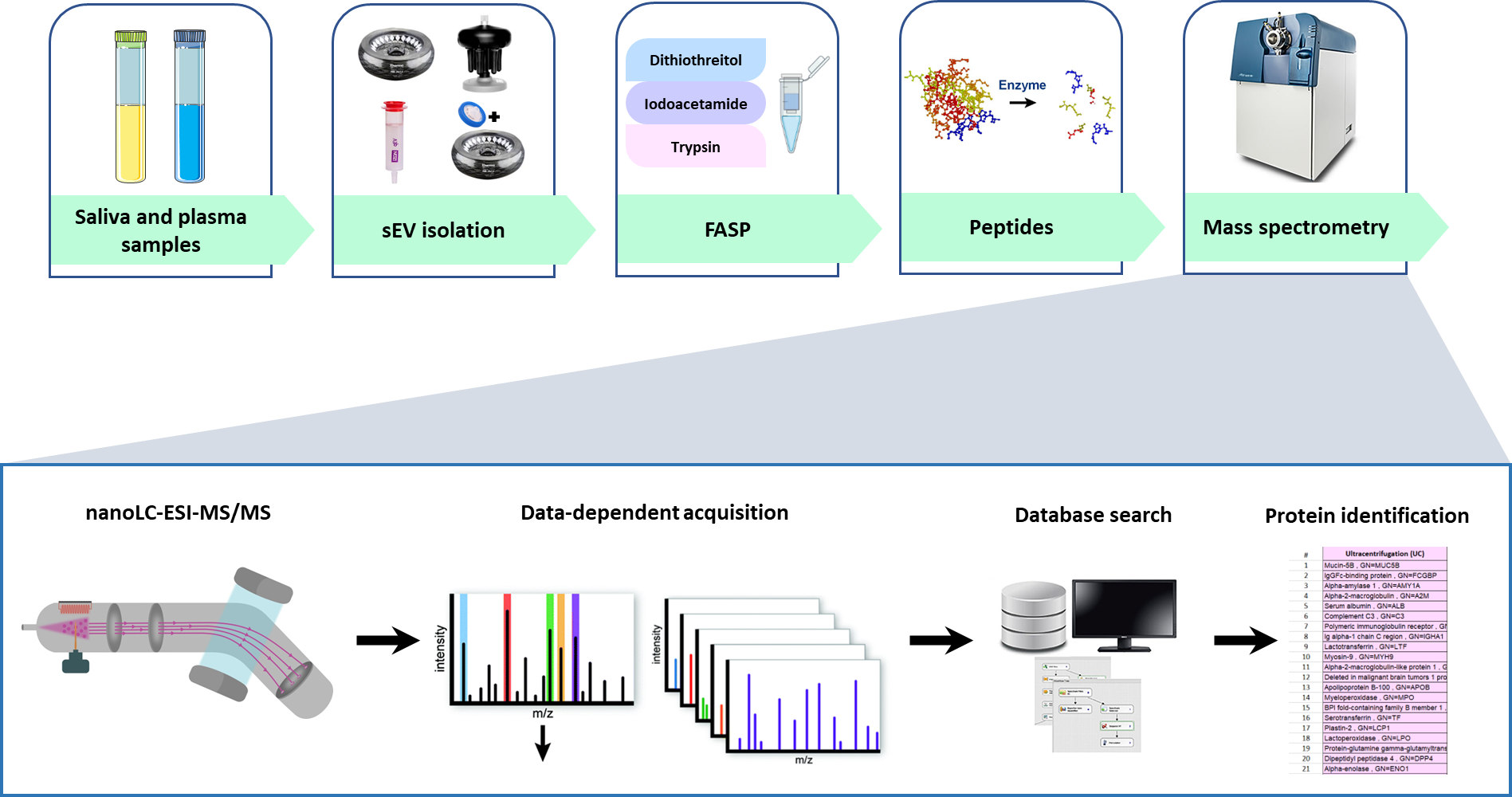


Figure S4. The study workflow for proteomics analysis. After sEV isolation, a total of 12.5μg of protein samples was aliquoted into low-binding tubes (Eppendorf, Hamburg, Germany) and processed using a filter-aided sample preparation (FASP) protocol within Microcon-30 kDa filter units (Millipore). Briefly, samples were reduced using dithiothreitol, alkylated using iodoacetamide and digested with trypsin. Peptides were desalted using STAGE tips containing SCX membranes and loaded onto nanoLC-ESI-MS/MS. Data-dependent acquisition (DDA) was performed on TripleTOF® 5600+ (SCIEX). The spectra of peptide ion fragmentations were collected and saved in wiff format (SCIEX). ProteinPilot was used to process MS data files and the protein lists were exported to .xls files.
